# Prevalence of Antinuclear Antibodies in Patients With Coronary Artery Disease: A Scoping Review

**DOI:** 10.7759/cureus.78915

**Published:** 2025-02-12

**Authors:** Manisha Antony, Christina Thymalil, Stephanie Nagy, Kayvan Amini, Marc M Kesselman

**Affiliations:** 1 Rheumatology, Nova Southeastern University Dr. Kiran C. Patel College of Osteopathic Medicine, Davie, USA; 2 Cardiology, Nova Southeastern University Dr. Kiran C. Patel College of Osteopathic Medicine, Davie, USA

**Keywords:** ana titers, antinuclear antibody, atherosclerosis, autoimmune disease, coronary artery disease, coronary artery ectasia

## Abstract

Coronary artery disease (CAD) is one of the most common causes of death worldwide. CAD is characterized by atherosclerosis and inflammation of the intima layer in blood vessels. Autoimmune diseases have a well-established association with CAD, which is accredited to the role immune modulators play in atherogenesis and autoimmune disease. Antinuclear-antibody (ANA) is an immunoglobulin that attacks nuclear and cytoplasmic components within cells, serving as a biomarker for many autoimmune conditions. This study evaluates whether ANA titers serve as an independent risk factor for CAD and its clinical manifestations. A scoping review was conducted using the following databases: CINAHL (Cumulative Index to Nursing and Allied Health Literature), Embase, ProQuest, OVID Medline, and PubMed. The search terms “Coronary artery disease” OR “Coronary disease” OR “Coronary aneurysm” AND “Antibodies, Antinuclear” were used to search for articles from 2000 to 2024. The initial search identified 442 articles and a total of 13 articles were included in the final results for data extraction. After analysis, 329 patients with CAD were found to have positive ANA titers, where a titer greater than 1:40 was considered positive. Among these patients, the most prevalent titer was 1:160 (0.31, n=103). Confounding rheumatological factors were evaluated: anti-cardiolipin IgG and IgM were the second most common antibodies (0.39, n=130) (0.37. n=123). Furthermore, a strong positive association between ANA titers and the number of stenotic coronary vessels was seen in the coronary artery ectasia (CAE) population. Hypertension was the most frequently observed comorbidity. Although smoking and being a male individual is a common risk factor for CAD, it was found that ANA titers were most prevalent in female nonsmokers. Results indicate that positive ANA titers could be an independent risk factor for CAD in patients without established autoimmune disease. This is evidence, particularly among patients with CAE, that the severity of ectasia in the coronary vessels is correlated with the strength of ANA titers. This investigation suggests that patients with positive ANA titers should undergo preliminary cardiovascular screening. Further research is needed to isolate ANA from traditional risk factors of CAD and to explore the potential use of ANA titers as a screening tool for CAD.

## Introduction and background

**Figure 1:**
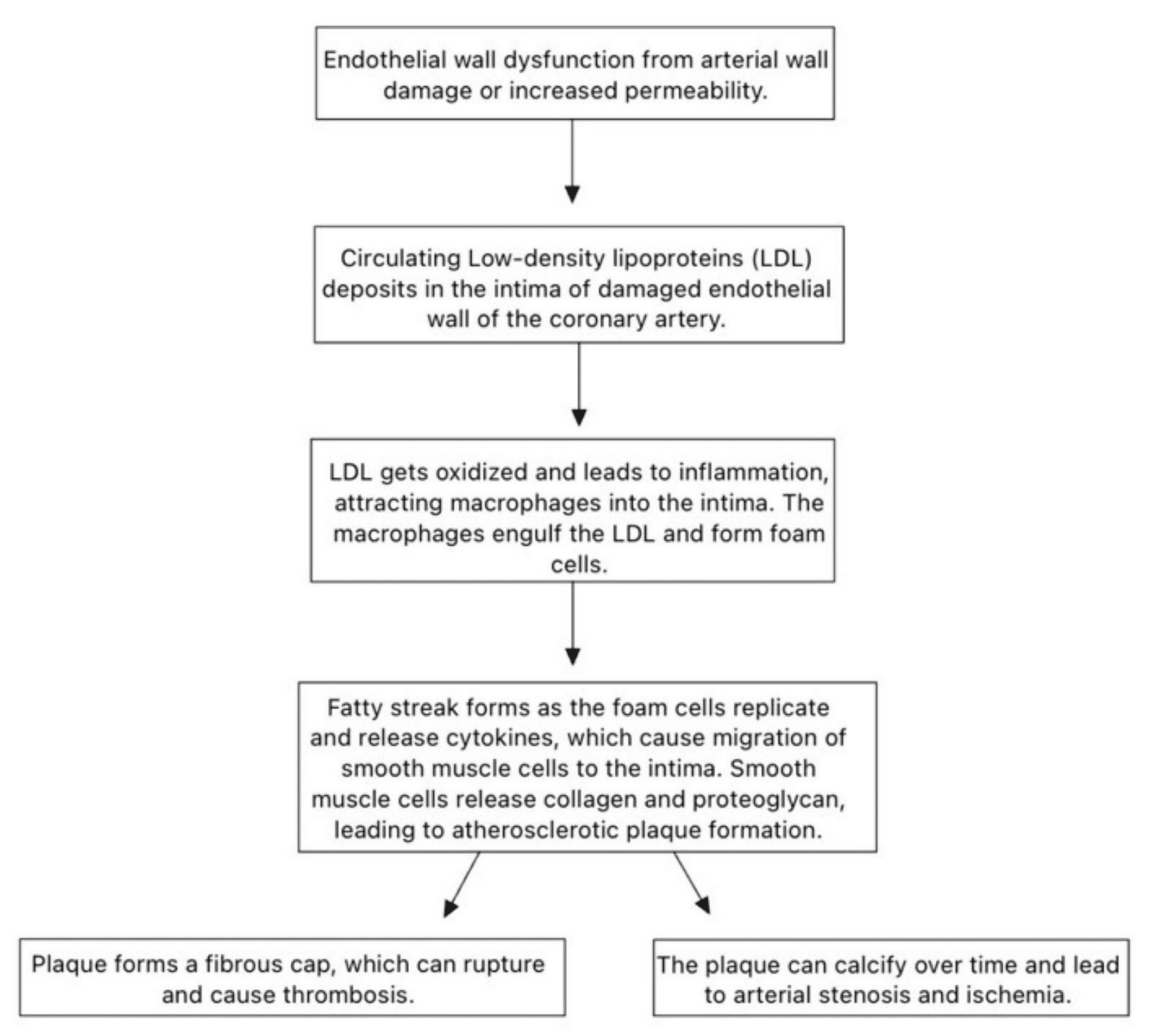
Pathophysiology of coronary artery disease. Image credit: Manisha Antony.

Coronary artery disease (CAD) is a cardiovascular disease that is prevalent in one in 20 adults, mainly men over the age of 65. CAD occurs due to damage to the blood vessels that leads to occlusion or atherosclerosis of the coronary arteries. Atherosclerosis occurs in the intima layer, which is the innermost layer of the blood vessels that consists of tunica intima, media, and externa. The development of CAD follows the step-wise progressive process that begins with lipoproteins penetrating endothelium, depositing in the intima layer, and undergoing oxidation [1]. These oxidized lipoproteins then attract leukocytes into the intimate layer and macrophages form foamy cells. This creates a fatty streak that causes the proliferation of smooth muscle cells, eventually forming atherosclerotic plaque that obstructs blood flow. The final lesion consists of a fibrous cap with an overlying lipid-rich core [1]. Pathophysiology can be visualized in Figure 1. If left untreated, CAD can be life-threatening and lead to stroke, heart failure, and myocardial infarction.

The traditional risk factors for CAD can be divided into two groups: modifiable and nonmodifiable. Modifiable risk factors include hypertension (HTN), hyperlipidemia (HLD), and diabetes. Patients with HTN who have a systolic blood pressure (BP) >140 mmHg, diastolic BP <70 mmHg, or increased pulse pressure are at an elevated risk for developing CAD [2]. This is due to high blood pressure (BP) and decreased myocardial perfusion exerting force on the arterial wall, leading to arterial wall stress and stiffness [3]. This stress leads to wall damage or endothelial dysfunction, such as vascular remodeling, and increased vascular resistance contributes to plaque formation [3]. Patients with elevated low-density lipoprotein (LDL) levels and decreased high-density lipoprotein (HDL) levels are at risk for developing coronary atherosclerosis as lipids play a major role in the development of plaques. Studies show that reducing cholesterol levels can stabilize plaques, making plaque rupture less likely and improving endothelial function [4]. Patients with A1C >7% are at risk for CAD due to higher coronary artery calcium (CAC) scores and faster progression of atherosclerosis [5]. CAC measures the amount of calcium in the arteries, which indicates the degree of calcified plaque in the arterial wall. This occurs as hyperglycemia can cause hypertension, hyperlipidemia, increased restenosis, and platelet dysfunction, which are all contributing factors to CAD [5]. Moreover, nonmodifiable risk factors include age > 35, male gender, family history, and ethnic group consisting of Blacks, Hispanics, Latinos, and Southeast Asians [6]. 

It is crucial for both patients and clinicians to be aware of the risk factors of CAD for early diagnosis, prevention, adequate lifestyle changes, and therapeutic treatment in order to decrease progression or even prevent CAD. Patients with systemic autoimmune diseases have a great risk of developing cardiovascular disease due to inappropriate inflammation that progresses throughout all body systems. An important marker of autoimmune disease is antinuclear-antibody (ANA) titers, which is an immunoglobulin that attacks nuclear and cytoplasmic components within cells, serving as a biomarker for many autoimmune conditions such as rheumatoid arthritis, systemic lupus erythematosus (SLE), Sjogren’s syndrome, scleroderma, and polymyositis [7]. However, about 25% of the general population have positive ANA titers, while only a small fraction of these individuals will develop autoimmune disease [8]. While there is an established association between autoimmune disease and cardiovascular disease, as shown there still is a larger number of the population that is silently ANA+ that may also have the same or similar risk of developing premature atherosclerosis and CAD. One potential cause of the presence of ANA in CAD patients without autoimmune disease is oxidative stress caused by reactive oxygen species (ROS). Oxidative stress can lead to antigenic structural changes in macromolecules causing the immune system to recognize the macromolecules as neoantigens leading to molecular mimicry [9]. Molecular mimicry occurs when immune systems recognize self-molecules as foreign and trigger an immune response by B cells and T cells resulting in inflammation and autoimmunity. For instance, oxidative stress to mitotic spindle antigens, NuMA and NuMA2, can form ANA known as anti-NuMA1 and anti-NuMA2 [10].

Additionally, recent evidence has strongly supported the association between atherosclerosis and autoimmunity; animal-model studies have shown that atherosclerotic-prone mice are more likely to have abnormal immune function, higher levels of anti-oxidized LDL autoantibodies and anti-cardiolipin antibodies, as well as, increased activation of polyclonal B cells within spleen tissue [11]. Furthermore, atherosclerotic-prone mice are more likely to have higher levels of ANA titers, IgG and anti-dsDNA titers, and pathognomonic titers of several autoimmune diseases, such as SLE, which suggests abnormal immunity in atherosclerotic pathogenesis [11]. Wang et al. suggest that the down-expression of the toll-like receptors 4 (TLR4) pathway and Bax in spleen tissue induced by lipopolysaccharide (LPS), which is the central process of atherosclerosis, may also lead to increased ANA titers [11]. LPS is a ligand of TLR-4, which inhibits apoptosis of monocytes, neutrophils, macrophages, and cardiocytes, while Bax, is a protein in the Bcl-2 family which competitively inhibits Bcl-2 and promotes apoptosis of lymphocytes [11].

Inappropriate inflammation is key in the development of both atherosclerosis and ANA titers associated with autoimmunity, thus the prevalence of ANA titers in patients with coronary artery disease is an important risk factor with great clinical relevance. This paper will explore the prevalence of ANA titers in patients with established CAD and their clinical relevance.

## Review

Methods

Search Strategy

A scoping review was conducted using the following databases: CINAHL (Cumulative Index to Nursing and Allied Health Literature), Embase, ProQuest, OVID Medline, and PubMed. Boolean operators “AND” and “OR” were used alongside keywords “Coronary artery disease” OR “Coronary disease” OR “Coronary aneurysm” AND “Antibodies, Antinuclear” to conduct the search. The article search was limited to those in the English language published between 2000 to 2024. The screening was conducted in a hierarchical approach where initial screening was based on the relevancy of the title and abstract, and secondary screening was based on the full manuscript. Articles without full-text availability through institutional access from the Nova Southeastern University library database were not included. 

Selection Criteria

Inclusion criteria consisted of patient populations over the age of 18 with clinical evidence of coronary artery disease, positive ANA titers, and articles that were peer-reviewed. Eligible study designs included case reports, randomized control trials, case-control studies, and prospective/retrospective studies. Exclusion criteria included patients with established autoimmune disease diagnoses and articles that were scoping reviews, editorials, opinion pieces, abstracts, or posters. The final articles were evaluated using the Joanna Briggs Institute critical appraisal checklists to assess reliability, quality, and efficacy [12]. All 13 articles underwent evaluation and were found to meet appropriate criteria as seen in Table 1 and Table 2.

**Table 1:** Joanna Briggs Institute critical appraisal of case report studies.

	Were patient's demographic characteristics clearly described?	Was the patient's history cleared described and presented as a timeline?	Was the current clinical condition of the patient on presentation clearly described?	Were diagnostic tests or assessment methods and the results clearly described?	Was the intervention(s) or treatment procedure(s) clearly described?	Was the post-intervention clinical condition clearly described?	Were adverse events (harms) or unanticipated events identified and described?	Does the case report provide takeaway lessons?	Overall appraisal
Li et al. [13]	Yes	Yes	Yes	Yes	Yes	Yes	Yes	Yes	Include
Lin et al. [14]	Yes	No	N/A	Yes	Yes	Yes	No	Yes	Include
Murai et al. [15]	Yes	Yes	Unclear	Yes	Yes	Yes	Yes	Yes	Include

**Table 2:** Joanna Briggs Institute critical appraisal of cohort studies. N/A: Not applicable.

	Were the two groups similar and recruited from the same population?	Were the exposures measured similarly to assign people to both exposed and unexposed group?	Was the exposure measured in a valid and reliable way?	Were the confounding factors identified?	Were strategies to deal with confounding factors stated?	Were the groups/participants free of the outcome at the start of the study (or at the moment of exposure)?	Were the outcomes measured in a valid and reliable way?	Was the follow-up time reported and sufficient to be long enough for outcomes to occur?	Was follow-up complete, and if not, were the reasons for loss to follow-up described and explored?	Were strategies to address incomplete follow-up utilized?	Was appropriate statistical analysis used?	Overall appraisal
Xanthopoulou et al. [16]	Yes	Yes	Yes	Unclear	Unclear	No	Yes	Yes	Yes	No	Yes	Include
Meroni et al. [17]	Yes	Yes	Yes	Yes	Yes	Yes	Yes	Yes	Yes	Yes	Yes	Include
Mazurek et al. [18]	Yes	Yes	Yes	Yes	Yes	No	Unclear	Yes	Yes	Yes	Yes	Include
Sedaghat et al. [19]	Yes	Yes	Yes	Yes	Yes	Yes	Yes	Yes	Yes	Yes	Yes	Include
Chalikias et al. [20]	Yes	Yes	Yes	No	No	Yes	Yes	Yes	Yes	N/A	Yes	Include
Edwards et al. [21]	No	Yes	Yes	Yes	No	No	Yes	N/A	N/A	N/A	Yes	Include
Grainger et al. [22]	Yes	Yes	Yes	No	No	Yes	Yes	Yes	Yes	N/A	Yes	Include
Solow et al. [8]	N/A	Unclear	Yes	Yes	N/A	No	Yes	Yes	N/A	N/A	Yes	Include
Katritsis et al. [23]	Yes	Yes	Yes	Yes	No	Yes	Yes	Unclear	Yes	N/A	Yes	Include
Brusca et al. [24]	Yes	Yes	Unclear	Yes	Unclear	Yes	Yes	Yes	Yes	Yes	Yes	Include

**Figure 2:**
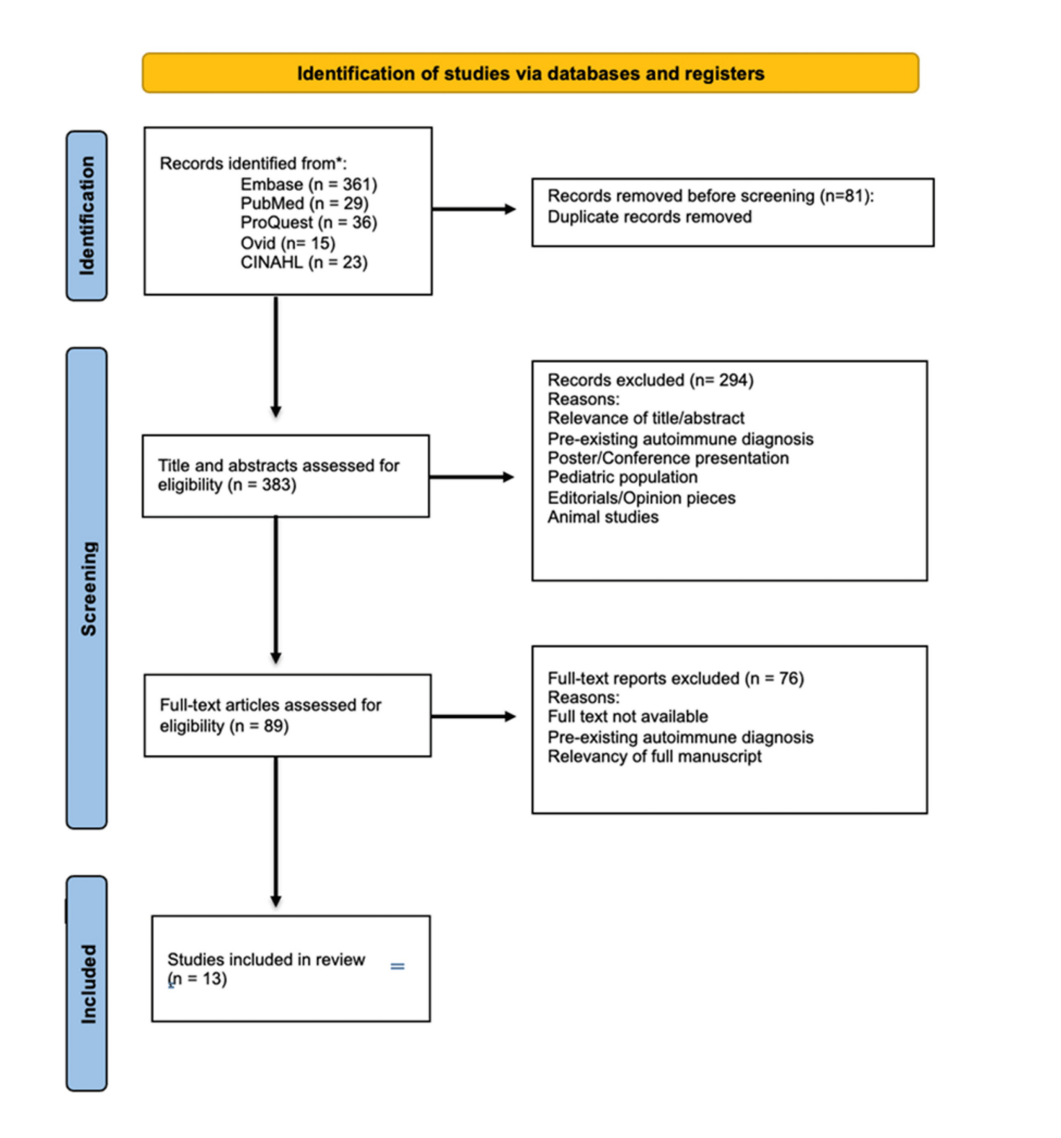
PRISMA flow diagram. PRISMA: Preferred Reporting Items for Systematic Reviews and Meta-Analysis.

A flow diagram of the selection criteria (Figure 2) was developed using the updated requirements outlined by the Preferred Reporting Items for Systematic Reviews and Meta-Analysis (PRISMA).

Results

The initial search using the specific keywords and five databases identified 442 articles. Following deduplication, 81 articles were excluded. A total of 383 articles were then screened based on title and abstract and 294 articles were excluded as they did not meet the eligibility criteria. Following this, 89 articles were screened based on full text, and a total of 13 articles were included in the final results for data extraction. The 13 articles were split into case studies and cohort studies for data extraction. Table 3 demonstrates the characteristics of the three case reports and Table 4 demonstrates the characteristics of the 10 cohort studies included in this scoping review.

**Table 3:** Characteristics of patients analyzed from the case reports. mmHg: millimeters of mercury; yo: years old; ANA: antinuclear antibodies; +: positive; ESR: erythrocyte sedimentation rate; mm/h: millimeters per hour; WNL: within normal limits; RCA: right coronary artery; LAD: left anterior descending; anti-RNP: antibodies to ribonucleoproteins; anti-Sm: antibodies to small nuclear ribonucleoproteins; anti-SSA: anti–Sjögren's-syndrome-related antigen A; SLE: systemic lupus erythematosus; CAD: coronary artery disease; mg: miligrams; IU: international unit; anti-Ro52: antibodies to Ro52 protein; LAD: left anterior descending; CATCH: coronary atherosclerosis T1-weighted characterization; HTN: hypertension; BMI: body mass index; IU/L: international unit per liters; mg/dL: milligrams per deciliter; MI: myocardial infarction; CK: creatine kinase; Hemoglobin A1C: glycated hemoglobin; anti-dsDNA: antibodies to double stranded deoxyribonucleic acid; LA; lupus anticoagulant; LDL: low-density lipoprotein; APS: anti-phospholipid syndrome; CRP: C-reactive protein.

Study	Year of publication	Country	Participant characteristics (age, sex)	Chief complaint	Pertinent history	Rheumatological comorbidities	Other comorbidities	Pertinent lab values	ANA titers	Lipid levels	Other + rheumatological lab values	Coronary angiogram results	Post-admission rheum diagnosis	Limitations	Home meds	In-patient medications
Li et al. [13]	2024	China	29 yo male	Intermittent strangulation and crushing chest pain for 10 days	Associated symptoms include shoulder and back pain, odynophagia and toothache. Pertinent negatives include no history of trauma, cough, fever, syncope or palpitations. Patient was a non-smoker with normal lipid panel.	None prior to admission.	None reported	Abnormal Q waves and a slight ST elevation in the inferior (II, III and aVF) limb leads. The patient's blood pressure was 133/85 mmHg and serum cardiac troponin T level was 0.517 ng/ml. The left ventricular ejection fraction was 59%. ESR was elevated at 36 mm/h.	1/1280	States lipid levels were WNL, patient was on rosuvastatin	anti‑nuclear ribonucleoprotein/Smith, anti‑Sjogren's syndrome A antibodies.	Long, extended, spiral‑shaped dissection of the RCA. No significant stenosis was observed in the left main artery, LAD artery or left circumflex artery.	SLE	Patient was diagnosed with SLE during his episode so difficult to infer only +ANA titer as reason for CAD.	None reported.	Aspirin (100 mg/day), clopidogrel (75 mg/day), rosuvastatin (20 mg/day), metoprolol succinate (47.5 mg/day) and low‑molecular‑weight heparin (5,000 IU) twice daily.
Lin et al. [14]	2021	China	60 yo female	1-year history of exertional angina	None reported.	None prior to admission.	None reported	ESR dropped from 70 to 8 mm/h 1 month later.	ANA positive reported	No lipid levels given.	anti-Ro-52, and anti-Sjogren syndrome A antibodies.	Spotty calcification in LAD and multiple severe stenoses in left main artery, LAD, circumflex artery, RCA with thickened wall.	coronary arteritis secondary to undifferentiated connective tissue disorder	Limited case presentation due to case report interest in CATCH.	Not reported.	Not reported.
Murai et al. [15]	2019	Japan	56 yo Japanese man	Acute chest pain	Episodes of fever and hypersensitivity to light when patient was 30 yo; history of cerebral infarcton at 54 yo which was treated with clopidogrel; while hospitalized patient had low grade fever but no sign of infection and erythema bilaterally on the face	None prior to admission.	HTN, smoker, underweight (BMI =17.9)	ST elevation in precordial leads suggesting acute anteroseptal MI, CK=. 1511 IU/L, Troponin= 1.4 ng/mL, Lactate dehydrogenase 454 IU/L, Hemoglobin A1c 6.5%, LDL=95 mg/dL.	1/1280	LDL=95 mg/dL	anti-dsDNA, positive direct Coombs test, lupus anticoagulant	Abrupt and total occlusion of the LAD artery	SLE, APS	Smoking is a confounding variable, does not reported other inflammatory markers such as ESR and CRP	Clopidogrel, HTN meds	Warfarin on hospital day 9, Aspirin, Clopidogrel

**Table 4:** Characteristics of patients from the cohort studies. CAE: coronary artery ectasia; CABG: coronary artery bypass graft; ANA: antinuclear anitbodies; +: positivity; RCA: right coronary artery; ESR: erythrocyte sedimentation rate; CRP: C-reactive protein; MI: myocardial infarction; anti-β2GPI-IgG: anti-beta-2 glycoprotein I immunoglobulin G; anti-β2GPI-IgM: anti-beta-2 glycoprotein I immunoglobulin M; CAD: coronary artery disease; STEMI: ST segment elevation myocardial infarction; aCL: anticardiolipin; LA: lupus anticoagulant; ELISA: enzyme-linked immunosorbent assay; anti-SSA: anti–Sjögren's-syndrome-related antigen A; anti-Ro52; anti-SSB: anti-Sjögren's syndrome type B antibodies; anti-Scl70: autoantibodies against topoisomerase I; ANCA: antineutrophil cytoplasmic antibodies; ACS: acute coronary syndrome; NSTEMI: non-ST segment elevation myocardial infarction; CSA: chronic stable angina; N/A: not applicable; HTN: hypertension: DM: diabetes mellitus; HDL: high density lipoprotein; LDL: low density lipoprotein; IHD: ischemic heart disease; RF: rheumatoid factor; ECG: electrocardiogram; ASCVD: atherosclerotic cardiovascular disease; EU: enzyme-linked immunosorbent assay units; CVD: cardiovascular disease; IgM: immunoglobulin M; AECA IgA: anti-endothelial cell antibodies of immunoglobulin A; pANCA: perinuclear anti-neutrophil cytoplasmic antibodies.

Study	Year of publication	Country	Type of study	Inclusion criteria	Exclusion criteria	Experimental group	Control group	Other group	Results	Titers ANA	Lipid levels	Limitations
Xanthopoulou et al. [16]	2021	Greece	Prospective observational cohort study	Patients with evidence of CAE on angiography from January 2019 to January 2020.	Patients with coronary aneurysm post coronary interventions, hx of CABG, or known autoimmune diagnosis.	39 patients consisting of 90% males, 54% with one ectatic vessel, and 46% with more than one ectatic vessel.	10 patients with normal coronary arteries	10 patients with atherosclerotic coronary artery disease without ectasia	Cut-off for positive titers were 1/160; 46% of CAE group was ANA+, 10% of control group was ANA+, 0% of atherosclerotic group was ANA+. Positive correlation between number of ectatic vessels involved and degree of ANA positivity. Right coronary artery was the most common artery involved.	In CAE group: 25.6% (1:160), 15.4% (1:320), 5.2% (1:640)	No lipid levels given.	Small patient population, hypothesis generating study, other inflammatory markers such as ESR, ferritin, CRP were not assessed.
Meroni et al. [17]	2007	Italy	Multicenter case-control study	Premenopausal women from January 1998 to December 2006 with history of MI before age of 45 and underwent coronary angiography during hospitalization. Needed to have blood sample collection between 3-12 months after MI.	No criteria given.	172 pre-menopausal female patients with history of MI before the age of 45.	172 healthy patients matched with the experimental group based on age, sex and geographical origin with no history of thromboembolic events.	50 unselected patients with MI consisting of 43 males and 7 females, and mean age of 53. Blood samples were taken within three weeks of acute coronary event.	ANA levels and anti-cardiolipin titers were not statistically significant compared to experimental vs control groups. Anti-b2GPI antibodies was statistically significant between the experimental vs control groups, levels were increased in the experimental group. No association between ANA and MI was found, but there was an association between anti-b2GPI antibodies and MI incidence. This study shows that anti-b2GPI IgG/IgM are independent risk factors for MI in young pre-menopausal women even without the presence of atherosclerotic lesions.	No titers given.	No lipid levels given.	Case-control design is a limitation for interpreting the confounding effect of pharmacological therapy and plasma levels of factors that may vary over time.
Mazurek et al. [18]	2016	Poland	Retrospective cohort study	Patients under the age of 45 with diagnosis of CAD who underwent myocardial revascularization. Blood samples were taken 1 month after procedure.	Patients with previously diagnosed autoimmune conditions, concomitant treatment with immunosuppressive therapy, thyroid disorders, chronic infections or malignancies.	39 patients under the age of 45 with history of CAD and myocardial revascularization. In this group, the most likely initial presentation of CAD was STEMI (19 patients, 48.7%). 87.1% of patients from the experimental group was treated percutaneously.	41 healthy volunteers consisting of 7 males and 34 females with no history or symptoms of CAD.	N/A	Levels of all types of aPL were significantly higher in CAD patients including aβ2GPI in both IgG and IgM, and aCL in both IgG and IgM. LA was also more frequent in CAD group. 13 patients had positive ANA via indirect fluorescence assay, however only one patient did the ELISA test to confirm positivity. This patient had significantly elevated SS-A/Ro-52, and slightly elevated SS-B and Scl-70 antibodies. 23 patients were ANCA positive.	No titers given.	No lipid levels given.	Titers not measured before myocardial revascularization process; all patients should have gotten ANA ELISA done, thus ANA assay cannot be confirmed.
Sedaghat et al. [19]	2014	Iran	Cross-sectional study	Patients with ischemic heart disease at Chamran Hospital from July to October 2013; Inclusion criteria included male patients who are undergoing coronary angiography.	Patients with valvular heart disease, any type of prior surgeries, trauma during prior months, cardiomyopathy, liver disease, renal failure, arthritis, malignancies, inflammatory diseases, and use of oral anticoagulant therapy.	70 patients with ACS which included STEMI, NSTEMI and unstable angina.	70 patients in CSA group which was characterized as deep, poorly localized chest or arm discomfort, reproduced by physical exertion or emotional stress, relived by nitroglycerin	N/A	Positive correlation between ANA levels and Gensini scoring (scored severity and extent of coronary stenotic lesions), but no relationship between Rheumatoid factor levels and Gensini scoring. When adjusting for cofounding variable, i.e., age, HTN, DM, hyperlipidemia, smoking, ANA remained independently associated with Gensini scoring in the ACS group.	No titers given.	No lipid levels given.	Small population, lack of female patients.
Chalikias et al. [20]	2023	Greece	Cross-sectional case-control study	Patients that underwent coronary angiography at the cardiac catheterization lab between 2019-2022 and were diagnosed with coronary artery ectasia.	Patients who developed a coronary aneurysm after coronary interventions and patients with a history of coronary artery bypass graft surgery, bicuspid aortic valve, a known diagnosis of autoimmune disease, current infection, or on medications that increase ANA titers	319 patients	90 patients without coronary ectasia consisting of 60 patients with normal coronary artery and 30 patients with atherosclerotic CAD.	N/A	4.28% prevalence of CAE with 75% of patients having HTN, 62% having hyperlipidemia, and 31% having DM. Half the population had CAE in 1 vessel while other half had >1 vessel involvement. 40% of patients with CAE had ANA+ titer and 20% of controls had ANA+ titer. 67% of the population with ANA+ titer had 3 vessel CAE, 30% had 2 vessel, 37% had 1 vessel, and 20% had no CAE. More ANA-positive titers correlated with increased severity of CAE as well.	11% of patients with CAE (1:80), 21% (1:160), 6% (1:320), 2% (1:640).	Total cholesterol: 175 mg/dL LDL: 109 mg/dL HDL: 45 mg/dL Triglyceride: 155 mg/dL	Other autoantibodies such as anti-cardiolipin and rheumatoid factor were not measured.
Edwards et al. [21]	2007	England	Retrospective cohort study	Patients born between 1930 and 1939 who lived in Hertfordshire in 1998-2003 and completed a questionnaire by HCS.	No criteria given.	567 men and 589 women between ages 59 and 71 years with data available for ischemic heart disease (IHD), rheumatoid factor, ANA, anti-cardiolipin antibody, and traditional risk factors.	N/A	N/A	ANA was positive in 10.8% of the men and 12.2% of women. Rheumatoid factor was positive in 16.2% of males and 12.4% of females. Anti-cardiolipin antibodies were positive in 24.2% of males and 19.7% of females. No association between IHD and ANA was found. 6.6% of males with ANA+ titers had IHD and 5.6% of females with ANA+ titers had IHD. 9.3% of males with positive rheumatoid factor had IHD and 8.9% of women with positive rheumatoid factor had IHD. 10.9% of males with positive anti-cardiolipin and 11.2% of females with positive anti-cardiolipin had IHD. Study found that rheumatoid arthritis is independent risk factor for IHD in men.	No titers given.	No lipid levels given.	Titers of ANA was not measured.
Grainger et al. [22]	2002	UK	Case-control study	All patients were from Papworth hospital with no diagnosis of autoimmune disease. First group (TVD) had patients with significant CAD (50%>) in all 3 coronary arteries with angina stable for 1 month without any MI in the past 3 months. Second group (NCA) had patients with chest pain, positive exercise ECG and normal coronary angiogram.	In NCA group, patients with HTN, DM, valvular disease, left ventricle hypertrophy. In experimental group, patients with angina not stable and those who had MI in the past 3 months.	40 subjects in TVD between ages 53 and 76 years.	N/A	30 subjects in NCA between 48 and 74 years.	In NCA group 17% were ANA+ and in TVD group 70% were ANA+. 4 subjects (1 in NCA and 3 in TVD) had positive anticentrosome antibodies. 23% of TVD and 10% of NCA had positive anticytoplasmic antibodies. This correlates with higher autoantibodies against cytoskeletal protein in patients with vascular disease.	Presence of ANA titers in all 70 subjects determined with 1:40 ANA titer. Median for NCA group was 1:160 titer and TVD was 1:320 titer. In TVD group, 2.5% (1:40), 10% (1:80), 15% (1:160), 17.5% (1:320), 20% (1:640), 2.5% (1:1280), and 2.5% (1:2560). In NCA, 6.7% (1:80), 3.3% (1/:60), 3.3% (1:640), and 3.3% (1:1280).	NCA group- Total cholesterol: 5.95 mmol/l LDL: 4.24 mmol/l HDL: 1.08 mmol/l Triglyceride: 1.2 mmol/l TVD- Total cholesterol: 6.17 mmol/l LDL: 4.49 mmol/l HDL: 0.78 mmol/l Triglyceride: 1.8 mmol/l	Small population.
Solow et al. [8]	2015	Not specified	Population-based cohort study	Patients without CVD and no autoimmune disease with immunosuppressive medication.	Patients with ANA>65 EU.	2803 participants	N/A	N/A	Higher ANA found in females, African American, participants with HTN, and nonsmokers. No difference in ANA based on age, DM, hypercholesterolemia, and metabolic syndrome. ANA also associated with all-cause mortality, cardiovascular death, and ASVCD. Over a median 9.4-year follow-up, 158 total deaths, 54 cardiovascular deaths, and 157 ASCVD events were recorded.	<1/160	No lipid levels given.	No specific titers were given.
Katritsis et al. [23]	2010	Europe	Case-control study	Patients with angiographic diagnosis of CAE according to standard criteria.	Patients with acute coronary syndrome, coronary aneurysm secondary to stent implantation, chronic or acute infectious disease, use of steroid or anti-inflammatory drugs within the last 3 months, renal failure and cancer.	475 patients had coronary angiogram and only 27 had CAE.	30 patients who were age, body mass index, sex, and CAD prevalence matched to the CAE group.	N/A	CAD was present in 63.3% of control and 63% of experimental group. ANA was positive in 43.3% of control group. In experimental group, there was 33.3% with ANA+ titers, 25.9% with anti-endothelial cell antibodies igM+, 37% with AECA igA+, 7.4% with pANCA+, 3.7% ACL igG+, and 11.1% acl igM+. ANA was not statistically significant when compared with both group. AECA igA is the only antibody that was statistically significant and it is associated with inflammatory disorders that affect vascular system. It is positive in up to 87% of patients with rheumatoid arthritis. It causes proinflammatory effects in patients with CAE by increasing inflammatory infiltration of monocytes and lymphocytes into media and adventitia by increasing plasma levels of IL-6, E-selectin, intercellular adhesion molecule-1, and vascular adhesion molecule-1.	Out of the 33.3% in experimental group that had positive ANA, 66.7% (1:160), 11.1% (1:320), 66.7% (1:640), and 66.7% (1:1280).	81.5% had total cholesterol > 240 mg/dL.	Small population size and only used data from 2009 coronary angiograms.
Brusca et al. [24]	2002	UK	Case-control study	Patients admitted to the department of medicine or intensive care unit at the hospital with MI, transient ischemic attack, and ischemic stroke	No criteria given.	139 patients consisting of 86 males and 53 females with mean age 64.8 years. 50 patients had MI, 60 patients had ischemic stroke, 29 patients had transient ischemic attack.	50 sex matched healthy individuals with mean age 55.8 years.	N/A	32% of patients with ischemic events and 26% of healthy subjects from control group had ANA+ titers. The difference was not statistically significant.	15.8% (1:40), 10.8% (1:80), 2.2% (1:160), 1.4% (1:320), and 1.4% (1:640).	No lipid levels given.	No other autoantibodies were looked at and sample was small as well.

*Findings* 

**Figure 3:**
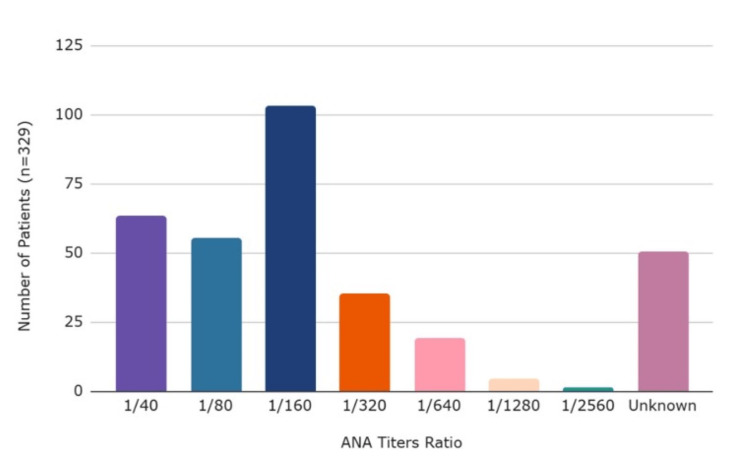
Prevalence of ANA titers in patients with ANA+ CAD. ANA: anti-nuclear antibodies; +: positive; CAD: coronary artery disease.

After analysis, 329 patients with CAD were found to have positive ANA levels with specific numerical values of titers recorded. Figure 3 illustrates the prevalence of ANA titers in patients with CAD [8,13-24]. Patients with an ANA titer greater than 1:40 were considered positive. Among these patients, the most prevalent titer was 1:160 (0.31, n=103).

**Figure 4:**
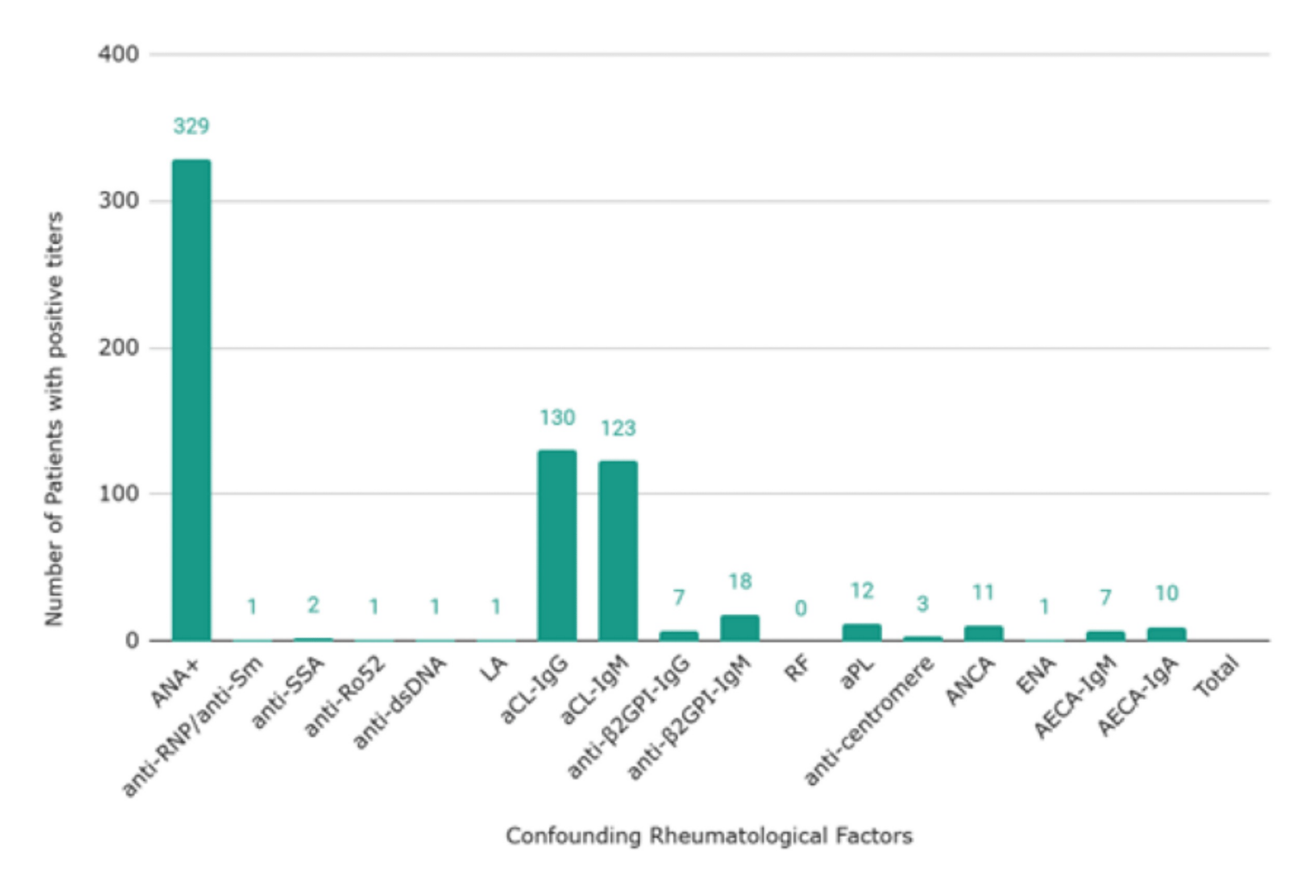
Confounding rheumatological factors seen in ANA+ CAD patients. ANA: anti-nuclear antibodies; +: positive; CAD: coronary artery disease; anti-RNP: antibodies to ribonucleoproteins; anti-Sm: antibodies to small nuclear ribonucleoproteins; anti-SSA: anti–Sjögren's-syndrome-related antigen A; anti-Ro52: antibodies to Ro52 protein; anti-dsDNA: antibodies to double stranded deoxyribonucleic acid; LA; lupus anticoagulant; aCL-IgG: anticardiolipin immunoglobulin G; aCL-IgM: anticardiolipin immunoglobulin M; anti-β2GPI-IgG: anti-beta-2 glycoprotein I immunoglobulin G; anti-β2GPI-IgM: anti-beta-2 glycoprotein I immunoglobulin M; RF: rheumatoid factor; aPL: anti-phospholipid antibodies; ANCA: antineutrophil cytoplasmic antibodies; ENA: extractable nuclear antigen; AECA-IgM: anti-endothelial cell antibodies of immunoglobulin M; AECA-IgG: anti-endothelial cell antibodies of immunoglobulin G.

Confounding rheumatological factors were evaluated as well. Figure 4 consists of other autoimmune antibodies found in patients with CAD and positive ANA titers. It was found that anti-cardiolipin (aCL) IgG (0.39, n=130) and IgM (0.37. n=123) were the second most common antibodies.

**Figure 5:**
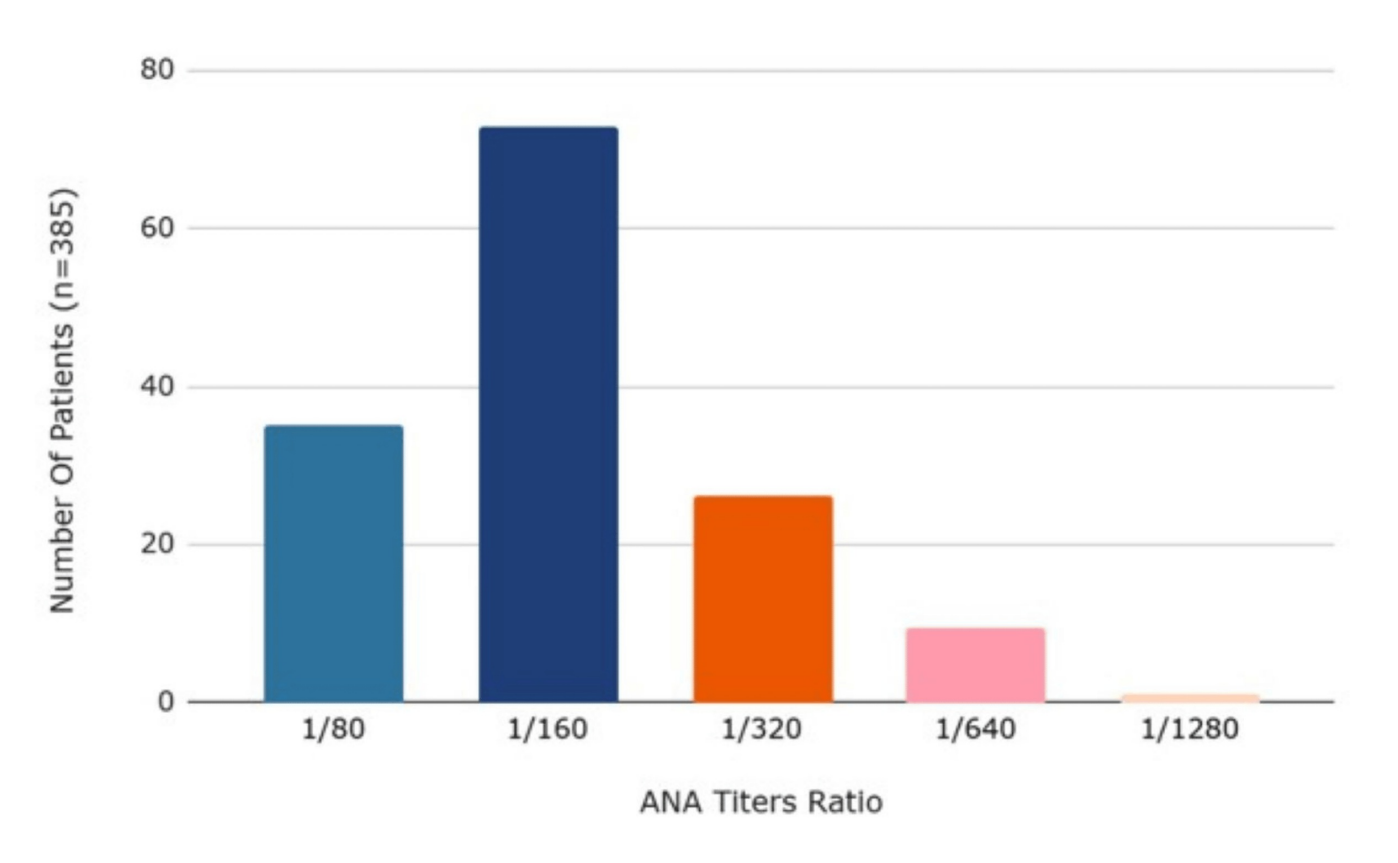
Prevalence of ANA titers in patients with CAE. ANA: anti-nuclear antibodies; CAE: coronary artery ectasia.

Further analysis showed a strong positive association between ANA titers and a specific subgroup of CAD known as coronary artery ectasia (CAE). Figure 5 demonstrates the prevalence of ANA titers in CAE [16,20,23].

**Figure 6:**
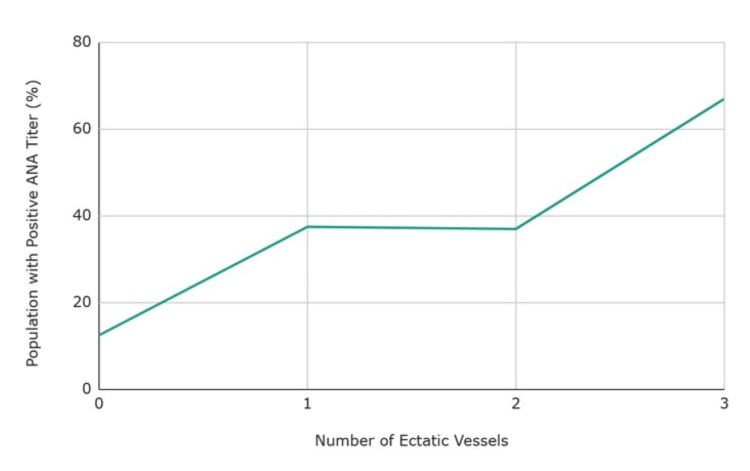
Correlation between positive ANA incidence and number of ectasic vessels. ANA: anti-nuclear antibodies.

The severity of ectasia in the coronary vessels is correlated with the strength of ANA titers. Figure 6 shows that most patients with positive ANA have all three coronary vessels involved in CAE [16,20,23]. 

Discussion

Role of Autoimmune Factors in CAD

Rheumatoid arthritis and other autoimmune diseases associated with positive ANA titers are known to cause systemic inflammation and immune dysregulation, leading to cardiovascular diseases such as endocarditis, myocarditis, pericarditis, and atherosclerosis [25]. Notably, even in the absence of autoimmune disease, autoantibodies are associated with an increased risk of cardiovascular disease. Roughly 25% of the population will test positive for ANA titers, yet very few of these individuals will ever develop an autoimmune disease, and the cardiovascular clinical implications of this in-between population with "benign autoimmunity" have yet to be studied [8]. This paper specifically explores how ANA levels play an independent role in the clinical manifestations of CAD. From the 10 cohort studies included in the results, five studies revealed an association between ANA positivity and CAD while the other 5 showed no significant difference between the experimental and control groups. Nevertheless, the proportion of patients with atherosclerotic disease that had positive ANA titers highlights the need for further investigation.

A study by Grainger et al. found that ANA titers of over 1:40 were present in 70% of their subjects who had coronary atherosclerosis of the three main coronary arteries while only 17% of the subjects with no evidence of atherosclerosis had positive ANA titers [22]. CAD begins with endothelial injury and plaque formation. ANA can play a role in the pathogenesis of CAD as ANA triggers immune response and inflammation when antibodies are produced against nuclear proteins. This results in immune system activation and immune complex deposition in blood vessels leading to organ injury and endothelial dysfunction [26]. Endothelial dysfunction contributes to plaque formation progression of atherosclerosis.

Patients with positive ANA titers that have CAD do not always present with the established risk factors of CAD such as male gender and smoker. A study by Solow et al. found that ANA titers were prevalent in females and non-smokers [8]. Their study also revealed that ANA positivity was associated with cardiovascular death so it is essential to monitor patients with positive ANA titers for cardiovascular disease, even if they do not meet the criteria for the traditional risk factors. Additionally, lipid levels were evaluated as confounding variables. A case study that followed a patient with CAD and positive ANA titers found that lipid levels were within normal limits, attributing atherosclerosis to ANA [15]. Another case-control study that showed the prevalence of ANA titers in CAD reported that lipids levels were elevated in both experimental and control groups [22]. Further research is necessary to determine the role of lipid levels in CAD influenced by ANA.

CAD is correlated with other autoimmune antibodies as well, specifically, antiphospholipid antibodies that consist of aCL and anti-β2-glycoprotein I antibodies (aβ2GPI) and are prothrombotic. A prospective study compared various autoantibodies in patients with cardiovascular diseases and healthy patients and found that only aCL IgG was significantly higher in patients with cardiovascular diseases, specifically CAD [27]. Another study compared autoantibodies to CAD and calcium score and identified significant differences in aCL, aβ2GPI, and oxLDL between healthy patients and patients with CAD [28]. However, there is no significant difference in calcium score between the two groups, which shows that autoantibodies can lead to CAD regardless of coronary calcification. These studies suggest the need for preliminary screening in patients with autoantibodies, specifically positive ANA titers.

ANA and CAE

CAE is a subgroup of CAD that causes diffuse or localized dilation of a coronary artery. The arterial segment can be at least 1.5 times dilated compared to a normal artery. CAE is found in 3-8% of patients through coronary angiography [29]. It can be acquired, congenital, or associated with inflammatory and connective tissue diseases such as Ehlers-Danlos syndrome, vasculitis, or Kawasaki disease [29]. Recent research has discovered an association of CAE with autoimmunity, especially ANA titers. From the 13 studies included in the results, three were focused on CAE with 383 patients having prevalence of ANA titers. A case-control study identified that patients with CAE had a 2.68-fold increase in ANA titers compared to patients with no CAE. Specifically, 40% of CAE patients had positive ANA titers and only 20% of the patients with no CAE had positive ANA titers [30]. Furthermore, ANA titers were most prevalent in patients with CAE involvement in all main coronary arteries. This suggests that ANA titers can play a role in the severity of ectasia.

ANA and Peripheral Artery Disease (PAD)

It is evident through research that CAD is correlated to autoimmune factors such as ANA titers. Nevertheless, it is necessary to evaluate the effect of ANA titers on other forms of atherosclerotic diseases such as PAD. PAD affects the abdominal aorta, iliac, and lower-extremity arteries and is prevalent in 12% of the population. It consists mainly of patients older than 70 years, or 50 years with smoking history or diabetes mellitus. It leads to symptoms such as claudication, ischemic rest pain and ulceration, revascularization, and limb loss [31]. A case-control study evaluated 121 patients with PAD for autoantibodies [32]. It was found that 38 of the patients tested positive for increased autoantibodies with ANA titers being the most common. There was no significant association between the severity of PAD and the concentration of autoantibodies [32]. When compared to CAD, ANA titers have much less effect on PAD.

Limitations

A key limitation of this study is the inconsistent patient information given between cohort studies and case reports. Although this paper argues an association between CAD and ANA titers is present, there is no evidence of causality which would require measurement of stenosis in coronary angiography or lipid levels in comparison to the strength of ANA titer. There is also no confirmation whether these patients have since been diagnosed with autoimmune diseases post-study publications. Furthermore, ANA positivity is a dynamic value that can increase during acute flare-ups in autoimmune disease thus one can assume ANA titers may change during acute episodes of CAD vs otherwise. In this study, ANA levels were taken during a wide range, and thus may be misleading.

## Conclusions

Results indicate that positive ANA titers could be an independent risk factor for CAD in patients without established autoimmune disease. This is evidence, particularly among patients with CAE, that the severity of ectasia in the coronary vessels is correlated with the elevation of ANA titers. As autoantibodies cause endothelial dysfunction, ANA may play a role in the pathophysiology of CAD. This investigation suggests that patients with positive ANA titers should undergo preliminary cardiovascular screening. Further research is needed to isolate ANA from traditional risk factors of CAD and to explore the potential use of ANA titers as a screening tool for CAD. Moreover, other autoantibodies such as Sjogren’s, topoisomerase, centromere should be evaluated for their influence on cardiovascular diseases.
